# RepCOOL: computational drug repositioning via integrating heterogeneous biological networks

**DOI:** 10.1186/s12967-020-02541-3

**Published:** 2020-10-02

**Authors:** Ghazale Fahimian, Javad Zahiri, Seyed Shahriar Arab, Reza H. Sajedi

**Affiliations:** 1 Bioinformatics and Computational Omics Lab (BioCOOL), Department of Biophysics, Faculty of Biological Sciences, Tarbiat Modares University, Tehran, Iran; 2 Department of Biophysics, Faculty of Biological Sciences, Tarbiat Modares University, Tehran, Iran; 3 Department of Biochemistry, Faculty of Biological Sciences, Tarbiat Modares University, Tehran, Iran

**Keywords:** Drug repositioning, Drug-diseases interaction, Biological network, Network integration, Machine learning, Breast cancer

## Abstract

**Background:**

It often takes more than 10 years and costs more than 1 billion dollars to develop a new drug for a particular disease and bring it to the market. Drug repositioning can significantly reduce costs and time in drug development. Recently, computational drug repositioning attracted a considerable amount of attention among researchers, and a plethora of computational drug repositioning methods have been proposed. This methodology has widely been used in order to address various medical challenges, including cancer treatment. The most common cancers are lung and breast cancers. Thus, suggesting FDA-approved drugs via drug repositioning for breast cancer would help us to circumvent the approval process and subsequently save money as well as time.

**Methods:**

In this study, we propose a novel network-based method, named RepCOOL, for drug repositioning. RepCOOL integrates various heterogeneous biological networks to suggest new drug candidates for a given disease.

**Results:**

The proposed method showed a promising performance on benchmark datasets via rigorous cross-validation. The final drug repositioning model has been built based on a random forest classifier after examining various machine learning algorithms. Finally, in a case study, four FDA approved drugs were suggested for breast cancer stage II.

**Conclusion:**

Results show the potency of the proposed method in detecting true drug-disease relationships. RepCOOL suggested four new drugs for breast cancer stage II namely Doxorubicin, Paclitaxel, Trastuzumab, and Tamoxifen.

## Background

Drug research and development is a complicated, time-consuming, and incredibly expensive process. Previous research reported that it often takes 10–15 years and approximately 1–3 billion dollars to develop a new drug and place it on the market [1–3]. Although such a huge amount of time and money is expending in this industry, the number of new Food and Drug Administration (FDA)-approved drugs reported annually remains low. So, in consideration of these challenges, discovering a new use for an existing drug, known as drug repositioning or drug repurposing, has been proposed as a solution for such a problem. The goal of drug repositioning is to identify new indications for drugs currently available in the market. Using such approaches can reduce the overall cost of commercialization and also bridge the gap between drug discovery and availability. In comparison to the traditional drug repositioning, which relies on clinical discoveries, computational drug repositioning methods can reduce the drug development timeline [4–6].

In recent years, different approaches are adopted for repurposing drugs, including network-based, text mining, machine learning, semantic inference-based methods. Recently, the network-based approach has attracted more attention and is widely used in computational drug repositioning due to the capability of using ever-increasing large-scale biological datasets such as genetic, pharmacogenomics, clinical and chemical data [7–10].

Networks are widely used in biology to comprehend and analyze the various connections in biological systems like protein–protein, gene–gene, and drug–target interactions. In such networks, nodes are representative of biological entities such as genes and proteins, while edges represent interactions between these components [11]. A variety of relationships can be introduced in a particular network at the same time. Moreover, quantitative information (weights) can be assigned to edges and nodes as well. Network-based drug repositioning methods can be divided into three classes regarding their main sources of biological data: (1) gene regulatory networks, (2) metabolic networks, and (3) drug interaction networks. Furthermore, a fourth category can be added to the above-mentioned classes, known as integrated approaches in which their data are provided simultaneously from multiple data sources. In gene regulatory networks, information about molecular perturbations, which occur because of drug administration or disease, can be captured via expression data. Metabolic networks give a different perspective. Nodes and edges in metabolic networks are representatives of the compounds and the metabolites. Drug–target interaction (DTI)-based prediction is one of the common repositioning methodologies. Indeed, many drugs frequently show additional targets than designed ones. For this reason, unintended novel usages can be shown through an effective and accurate prediction of drug targets. In addition to the previous strategies, there are other repositioning approaches based on several molecular networks. However, they show limited applicability [11–13].

In this study, we have proposed a network-based method for drug repositioning. Our method, RepCOOL, integrates various heterogeneous biological networks to obtain new drug-disease associations. The proposed method showed satisfactory performance in detecting drug-disease associations via stringent assessment procedures. Eventually, four new drugs were suggested for breast cancer.

## Method

**Fig. 1:**
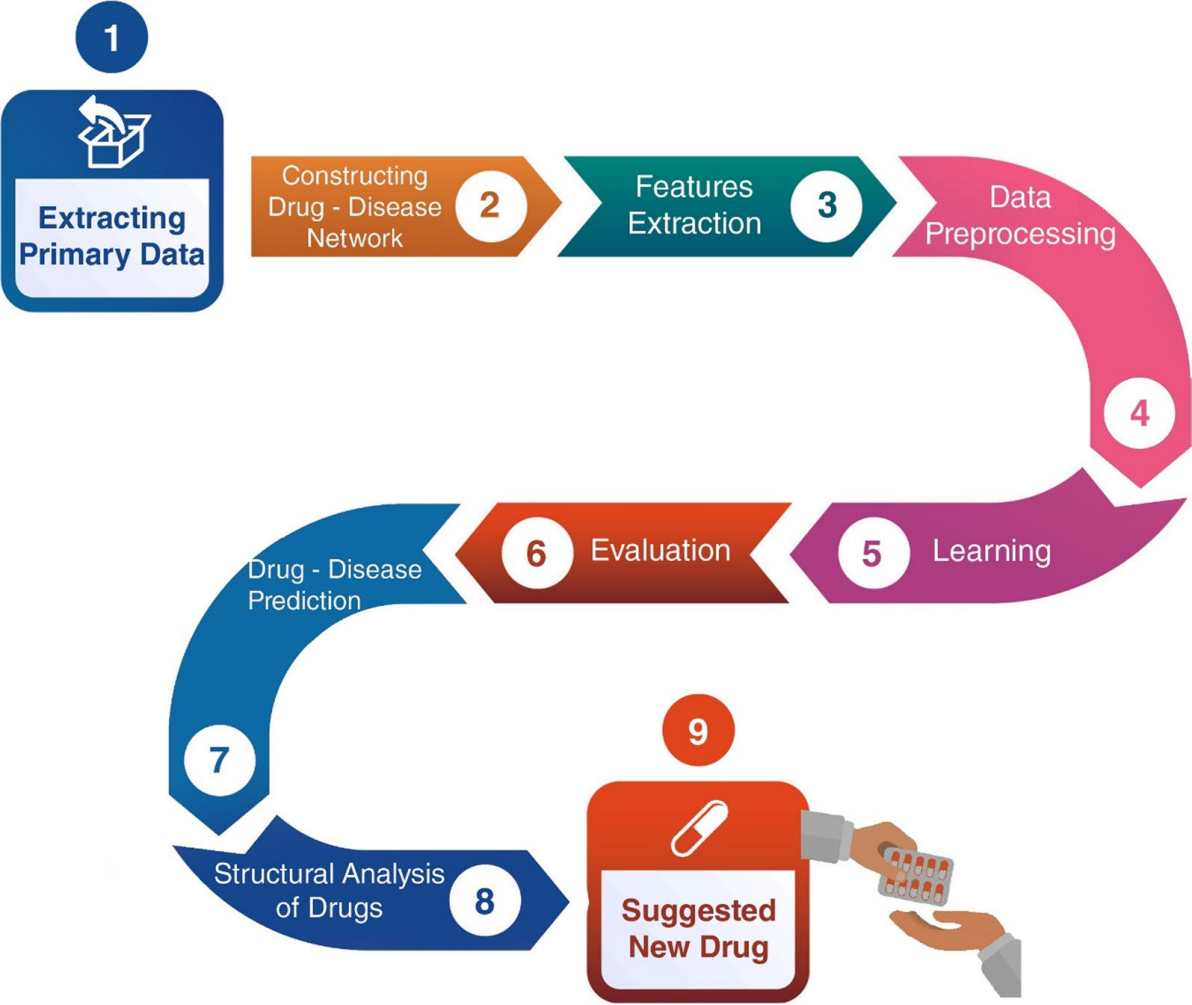
Schematic flowchart of the proposed drug repositioning method

Figure 1 shows an illustration of the proposed drug repositioning method. Detailed descriptions for each step are provided in the following subsections.

### Data sources

We constructed nine different drug-disease association networks using six primary networks constructed based on the publicly available database (Table 1). These six networks were categorized into four different groups according to their types of nodes: drug–gene interaction network (DRGN), disease-gene interaction network (DIGN), protein–protein interaction network (PPIN) and gene co-expression network (GCN).

**Table 1:** Primary data sources for drug-disease network reconstruction

Network type	Source database	Network details	URL address	References
DRGN	Drug bank	No. of drugs: 1497 No. of genes: 673 No. of interactions: 3509	https://www.drugbank.ca/	[14]
DIGN	CTD	No. of diseases: 3158 No. of genes: 47,740 No. of interactions: 26,047,815	http://ctdbase.org/	[15]
DIGN	OMIM	No. of diseases: 4552 No. of genes: 6175 No. of interactions: 6666	https://www.omim.org/	[16]
DIGN	DisGeNET	No. of diseases: 20,371 No. of genes: 17,068 No. of interactions: 561,107	http://www.disgenet.org/	[17]
PPIN	Intact	No. of proteins: 16,523 No. of interactions: 143,758	https://www.ebi.ac.uk/intact/	[18]
GCN	COXPRESdb	No. of genes: 24,442 No. of interactions: 12,485	http://www.COXPRESdb.org/	[19]

#### Drug–gene interaction network

DrugBank [14] database was used to construct the DRGN network. DrugBank provides comprehensive information about approved and investigational drugs, including UMLS-mapped, approved indications. This network consists of 3509 interactions between 1497 drugs and 673 genes.

#### Disease-gene interaction network

We also used three databases for three different disease-gene interaction networks (Table 1): The Comparative Toxic genomics Database (CTD) [15], Online Mendelian Inheritance in Man (OMIM) [16] and DisGeNET [17]. CTD contains manually curated information about gene-disease relationships focusing on comprehending the effects of environmental chemicals on human health. It includes about 26 million gene-disease associations (GDAs), between 47,740 genes and 3158 diseases. OMIM (Online Mendelian Inheritance in Man) is a complete collection of human genes and genetic phenotypes that are updated on a daily basis. OMIM includes 6666 gene-phenotype associations between 6175 phenotypes and 4552 genes. The DisGeNET database integrates human gene-disease associations from various expert-curated databases and text-mining-derived associations including Mendelian, environmental and complex diseases [17]. This network includes 561,107 GDAs, between 17,068 genes and 20,371 diseases, disorders, traits, and clinical or abnormal human phenotypes.

#### Protein–protein interaction network

We extracted protein–protein interaction (PPI) information from IntAct database [18]. IntAct provides a freely available database system and analysis tools for molecular interaction data. This network has 16,523 proteins and 143,738 protein–protein interactions.

#### Gene co-expression network

We constructed a gene co-expression network (GCN) using the COXPRESdb database [19]. This database measured the similarity of gene expression patterns during several conditions, such as disease state tissue types. COXPRESdb includes co-expression relationships for multiple animal species and is freely available on http://coxpresdb.jp/. The obtained GCN includes 12,485 interactions and 24,442 genes.

### Reconstructing new drug-disease networks via merging heterogeneous networks

**Fig. 2:**
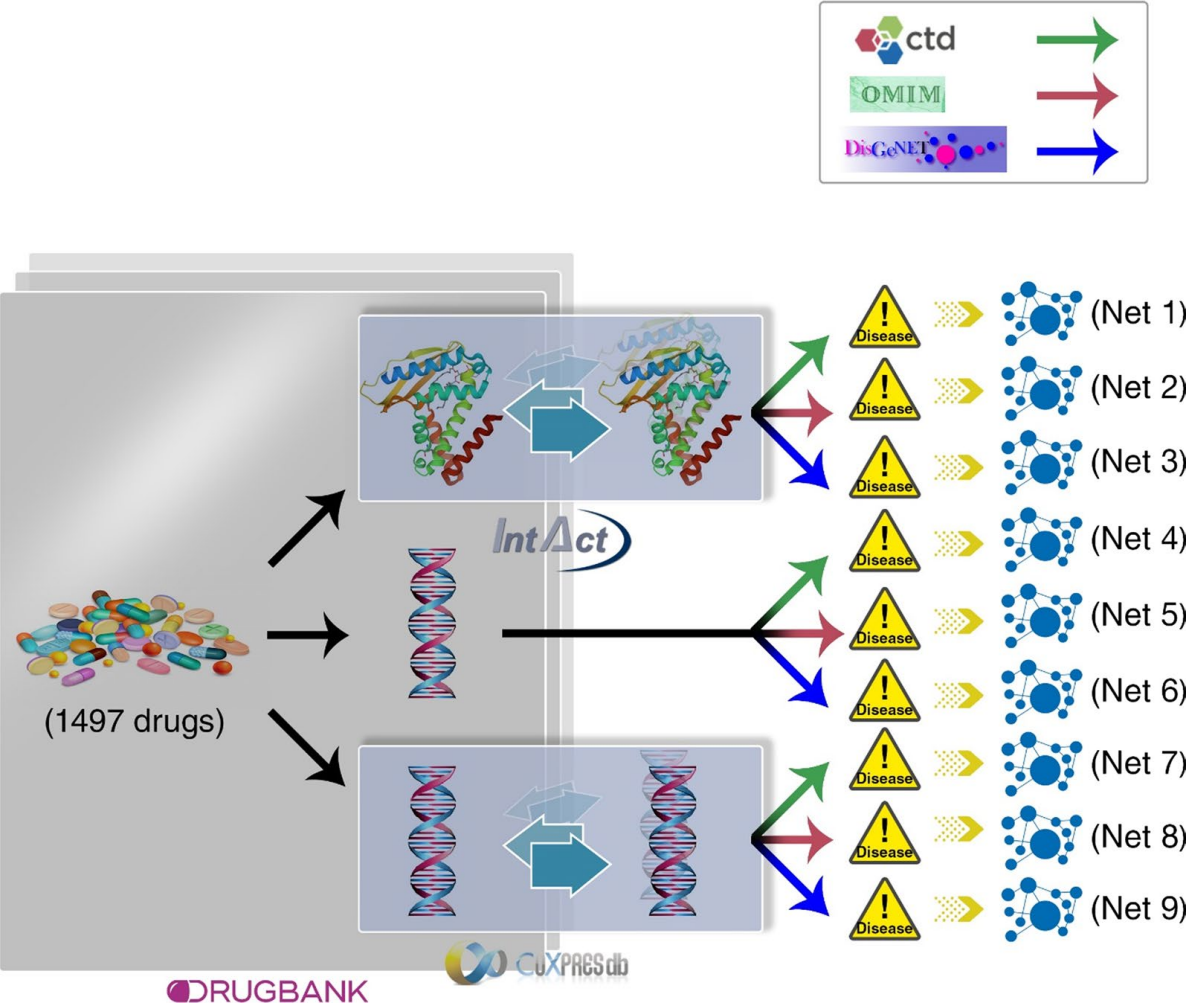
Schematic representation of reconstructing nine new drug-disease networks

We reconstructed nine new drug-disease networks using six primary networks. Figure 2 shows a schematic view of these networks. These nine networks have more than 9,400,000 drug-disease associations in total. Table 2 shows more details about these new drug-disease networks. One drug-disease interaction may be generated more than once in each network merging. So, the number of occurrences of a drug-disease interaction is considered as the weight of the interaction.

**Table 2:** Reconstructed drug-disease networks

Networks	Number of drug	Number of disease	Drug-disease association
Net1	1337	5854	4,129,617
Net2	1333	8540	397,108
Net3	1191	10,858	741,819
Net4	1208	11,934	8,256,300
Net5	164	2240	82,407
Net6	239	2306	92,299
Net7	94	2200	151,267
Net8	21	1013	329
Net9	17	468	812

### Drug-disease association prediction

#### Encoding drug-disease networks as feature vectors

For each drug-disease pair, weights of its corresponding interaction in the reconstructed drug-disease networks were considered as features. Therefore, each drug-disease pair was encoded as a 9-dimensional feature vector. In addition, to prevent the occurrence of the duplication in weighing the networks, the limitation of the initial datasets must be considered.

#### Machine learning methods

We used five different classifiers, including naïve Bayes (NB), random forest (RF), logistic regression (LR), decision tree (DT) and support vector machine (SVM). The implementations of these classifiers in Weka [20] software package was used for drug-disease association prediction. Weka is a java-based machine learning workbench, developed for machine learning tasks. Also, we used tenfold cross-validation for evaluating the predicted drug-disease associations.

For evaluating the performance of RepCOOL, we adopted four different measures (Table 3). These measures are based on the following four basic terms:

**Table 3:** Measures for assessing prediction performance

$Recall = \frac{TP}{TP + FN}$	Positive correctly predicted
$Precision = \frac{TP}{TP + FP}$	Positive predictive value
$Accuracy = \frac{{{\text{TP}} + {\text{TN}}}}{{{\text{TP}} + {\text{TN}} + {\text{FP}} + {\text{FN}}}}$	Correctly predicted
$F - measure = \frac{2 \times Presion \times Sensitivity}{Presion + Sensitivity}$	The harmonic mean of sensitivity and specificity

- True positive (TP): the number of drug-disease associations, which were correctly predicted.
- True negative (TN): the number of drug-disease pairs, which were correctly predicted as non-associated.
- False positive (FP): the number of unrelated drug-disease pairs, which were incorrectly predicted as associations.
- False negative (FN): the number of drug-disease associations, which were incorrectly predicted as non-associations.

We also used the area under the ROC curve (AUC) as another measure for assessing the proposed method.

#### Benchmark dataset

We used PREDICT [21], which is a well-known benchmark dataset in drug repositioning, to assess the strength of the proposed drug repositioning method. PREDICT dataset includes 1834 interactions between 526 FDA approved drugs and 314 diseases.

#### Cytotoxicity assay

Human cell line BT474 was cultured in recommended media in the presence of 10% fetal bovine serum (FBS) and penicillin–streptomycin antibiotics. Cell viability was characterized using a standard colorimetric MTT reduction assay. Briefly, 6000 cells were plated in each well of the 96-well plates with 100 µL medium, which includes 10% serum. After 24-h incubation, the cell was treated with several concentrations of tamoxifen (0–100 µM). After 48 h, the MTT (3-(4,5-dimethylthiazol-2-yl)-2,5-diphenyltetrazolium bromide) reagent (5 mg/mL in PBS) was added to each well, followed by incubation for 4 h at 37 **°**C with 5% CO_2_. After the incubation, the MTT crystals in each well were solubilized in 100 µL dimethyl sulfoxide (DMSO) incubation for 20 min at 25 **°**C, and the absorbance was read at 490 nm using a microplate spectrophotometer (µQuant, BioTek, USA).

## Results and discussion

### Performance evaluation of the proposed method

**Fig. 3:**
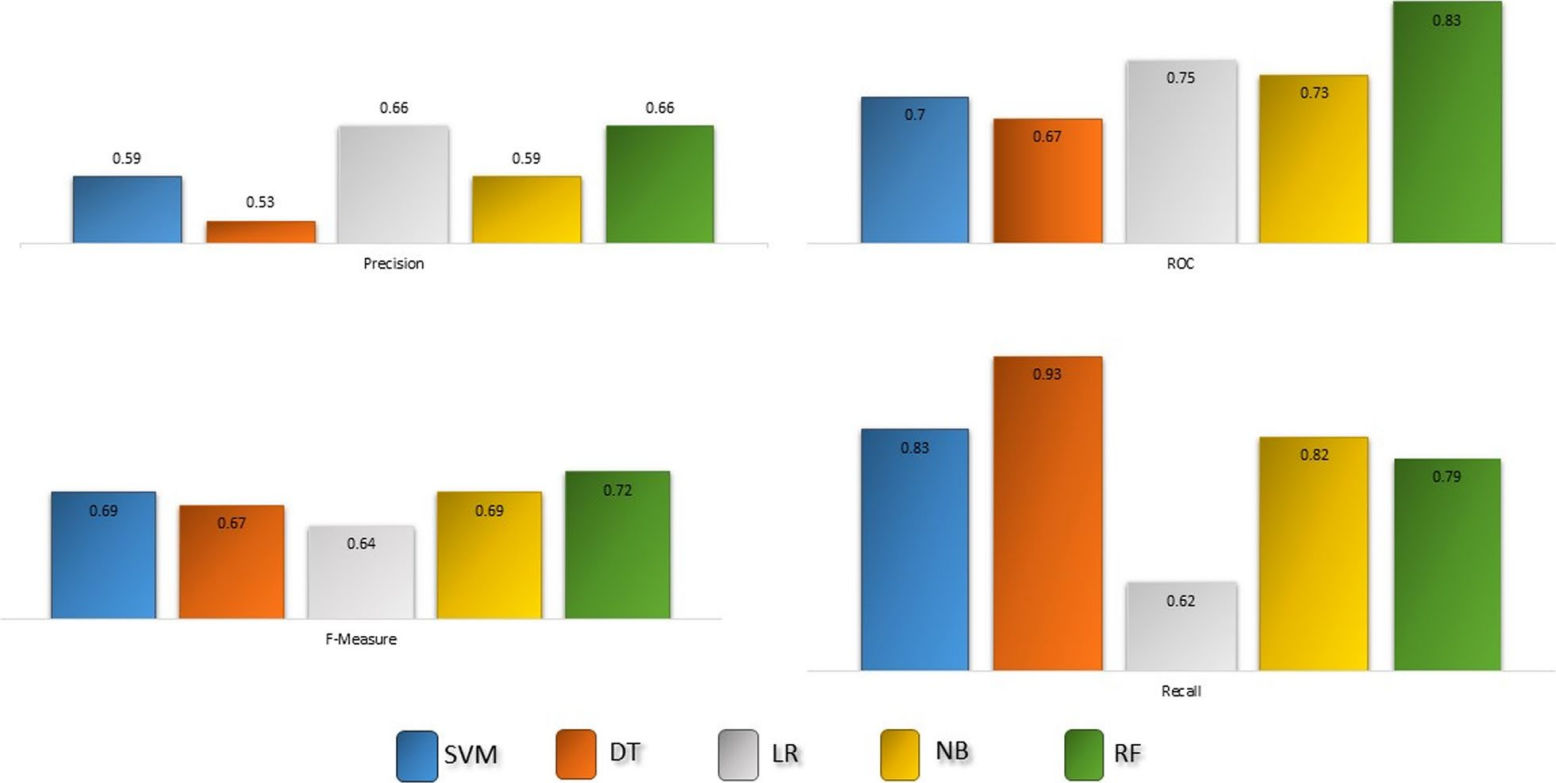
Performance of different classifiers in a tenfold cross validation procedure in PRIDICT dataset. Classifiers include support vector machine (SVM), decision tree (DT), linear regression (LR), naïve Bayes (NB) and random forest (RF)

Figure 3 shows the performance of five classifiers on the PREDICT dataset in a tenfold cross-validation experiment. As it was evident, the decision tree is the most sensitive classifier in detecting true drug-disease associations, but random forests have the best performance in terms of ROC. For all the classifiers, recall (sensitivity) is in a satisfactory range, which shows the ability to detect true drug-disease associations. However, precision is relatively low for almost all classifiers, which can result from some true drug-disease associations that have not been discovered or reported yet.

### Comparison with the other methods

**Fig. 4:**
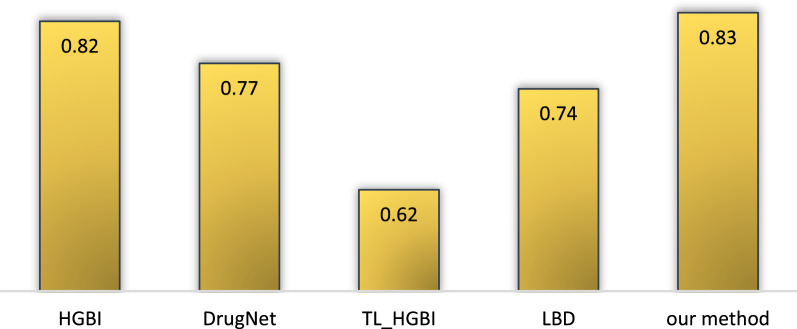
Performance comparison of RepCOOL with other methods in terms of AUC based on the obtained results in PREDICT dataset

Nearly all of the previously published studies only reported their AUC. As it has been shown in Fig. 4, the highest AUC of the five classifiers is 0.83, which outperforms HGBI [22], LDB [23], TL-HGB [24] and Drug Net [23] methods on PREDICT dataset.

### New repurposed drugs for breast cancer

**Fig. 5:**
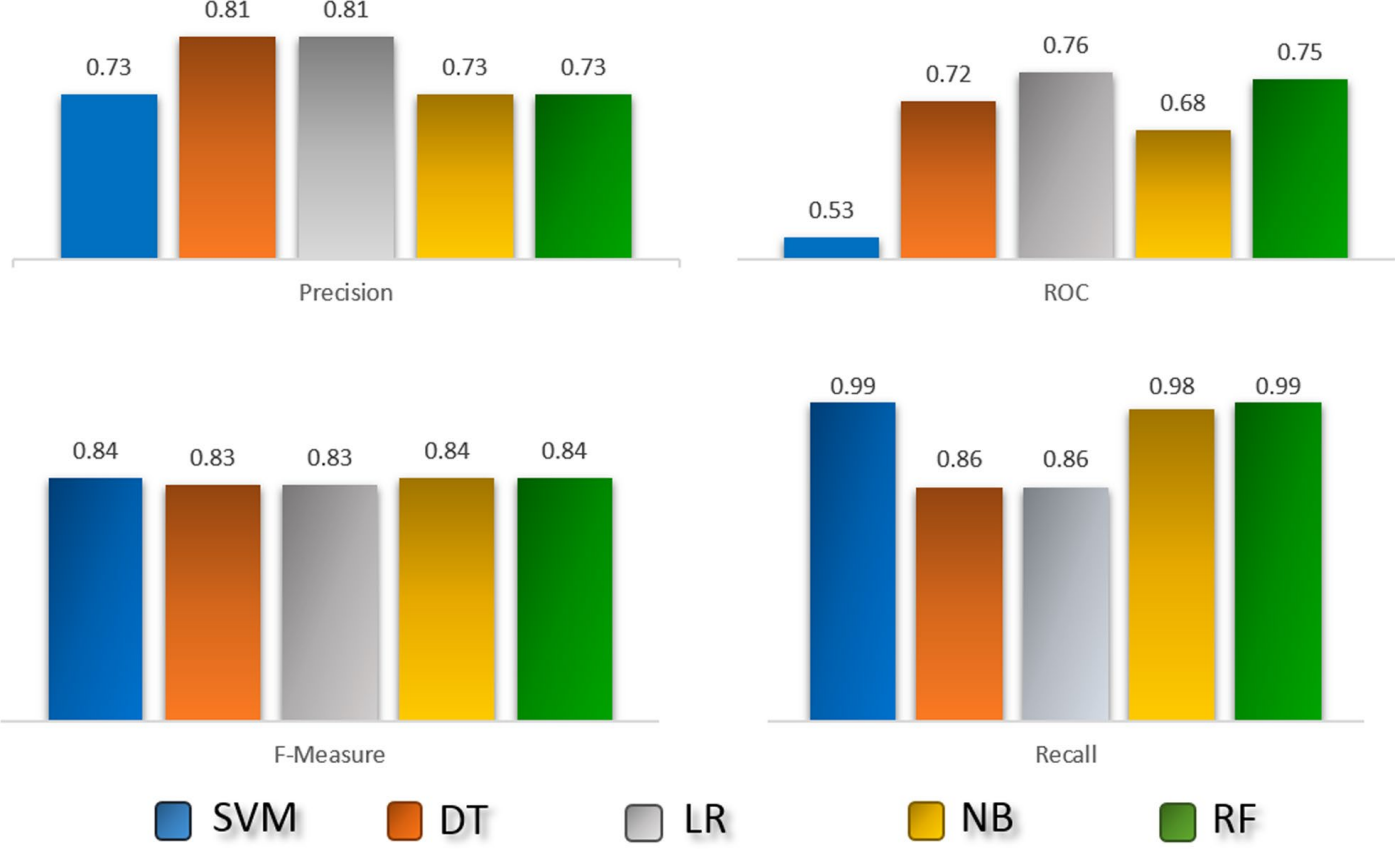
Performance of different classifiers in a tenfold cross-validation procedure in repODB dataset. Classifiers include support vector machine (SVM), decision tree (DT), linear regression (LR), naïve Bayes (NB) and random forest (RF)

Information contained in RepoDB [25] was exploited to obtain a list of new repurposed drugs for breast cancer. RepoDB includes a gold standard set of drug repositioning which failed or succeeded. The RepoDB dataset contains 6677 approved, 2754 terminated, 483 suspended, and 648 withdrawn drug-disease interactions. Withdrawn and suspended drug-disease associations have an annotation phase between phase 0 and phase 3. Therefore, these two types of drug-disease pairs have more potential to suggest a valid new drug repositioning rather than a random pair. Considering this fact, we trained the five classifiers using the approved and terminated data. Figure 5 shows the training performance of the classifiers. Then, the best performing classifier, according to the approved and terminated data, was used to predict new drugs for breast cancer. The most sensitive classifier, random forest (it detected 2283 true drug-disease interactions out of 2292), was used to do this end.

Using this classifier, four new drugs have been repurposed for breast cancer stage II. Table 4 shows the chemical structures of the drugs and their descriptions.

**Table 4:** Summary of function and structure of the repurposed drugs for breast cancer

Rank	Repurposed drugs	Current usages^a^	Structure
1	Doxorubicin	Treatment of leukemia, lymphoma, neuroblastoma, sarcoma, Wilms tumor, and cancers of the lung, breast, stomach, ovary, thyroid, and bladder	
2	Paclitaxel	Treatment of AIDS-related Kaposi sarcoma, advanced ovarian cancer, and certain types of breast cancer	
3	Tamoxifen	Treatment of the ovary, breast cancer, desmoid tumors and endometrial cancers	

^a^According to National Institutes of Health (NIH) (https: 2019, June) and Drug bank (https 2019, June)

### Analyzing the structural similarity between the three new repurposed drugs and previously FDA-approved drugs for breast cancer

**Fig. 6:**
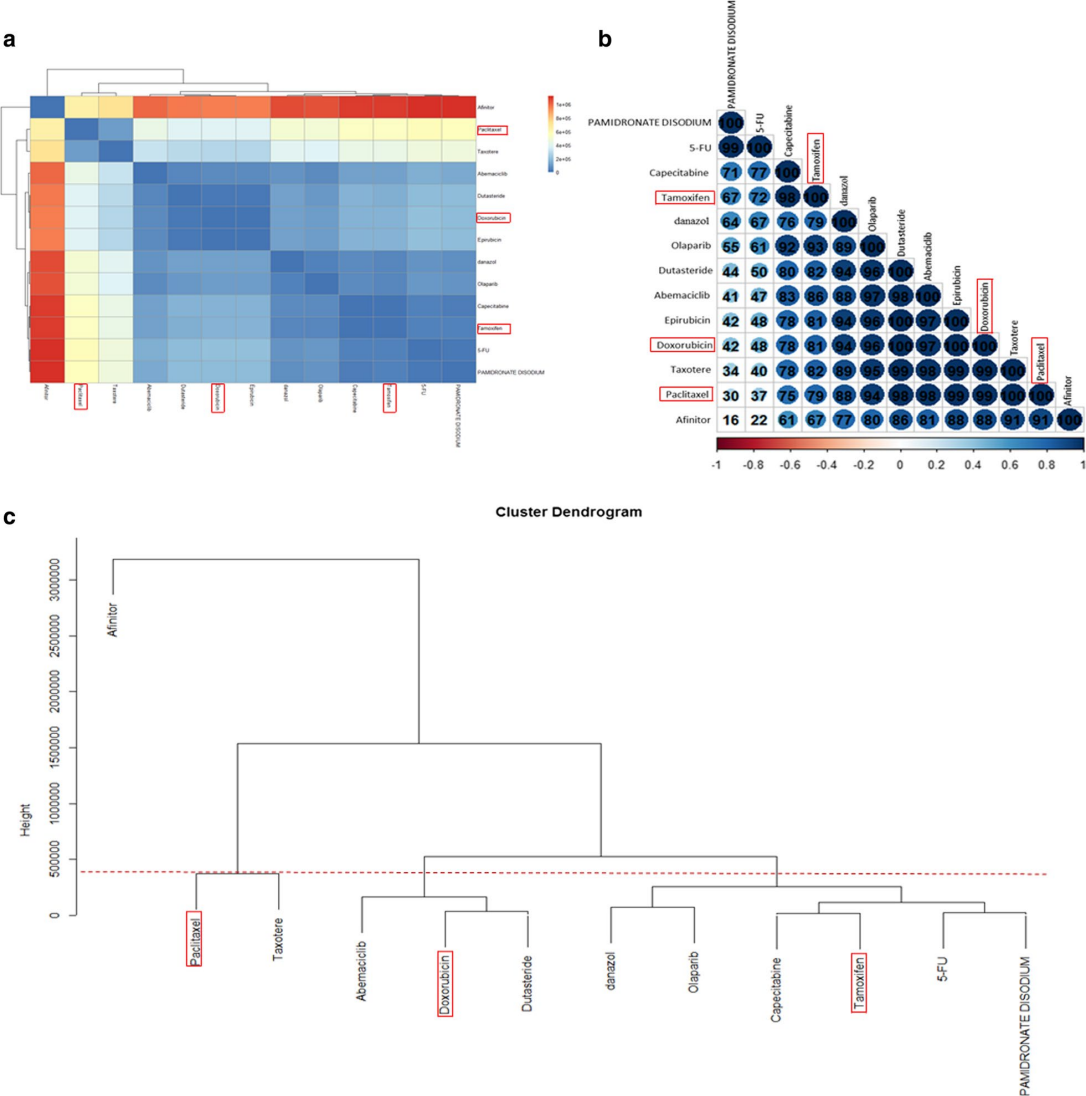
Structural relationship between the repurposed (highlighted by rectangles) and *FDA*-*approved drugs* for the treatment of breast cancer. **a** Heat map of the merged repurposed and *FDA*-*approved drugs* based on the distance matrix. **b** Heat map of repurposed and *FDA*-*approved drugs* based on the correlation matrix. **c** Cluster dendrogram of repurposed and *FDA*-*approved drugs* based on the distance matrix. The highest and the lowest structural correlation are indicated in blue and red, respectively

We also carried out a structural similarity analysis among the repurposed drugs and 10 FDA-approved which were small molecule drugs for breast cancer including 5-FU, Abemaciclib (Verzeino), Taxotere (docetaxel), danazol, Pamidronate Disodium, Tamoxifen, Doxorubicin, Paclitaxel, Epirubicin, Capecitabine, Dutasteride, Olaparib, Afinitor. Also, Trastuzumab is a recombinant DNA-derived humanized monoclonal antibody which was eliminated from our repurposed drugs due to its large structure (145,531.5 Da). Figure 6 shows the results of the structural similarity analysis. Structural similarity was computed based on 3014 structural features which were extracted using Dragon tool [26]. Figure 6a compares the structures of the drugs via a distance matrix, and Fig. 6b represents the correlation matrix of the structures computed with Pearson correlation coefficient (PCC). Also, Fig. 6c depicts the dendrogram of 13 drugs based on the obtained distance matrix. According to this dendrogram, there are four distinct clusters: cluster1 = {Paclitaxel, Taxotere}, cluster2 = {Doxorubicin, Dutasteride, Epirubicin, Abemaciclib}, cluster3 = {Afinitor} and cluster4 = {Pamidronate Disodium, Capecitabine, Tamoxifen, Olaparib, 5FU, Verzeino}. As results indicate, Paclitaxel, Doxorubicin and Tamoxifen have the most structural similarity with Taxotere (PCC = 100), Dutasteride, Epirubicin (PCC = 100) and Capecitabine (PCC = 98), respectively.

### Cell toxicity

**Fig. 7:**
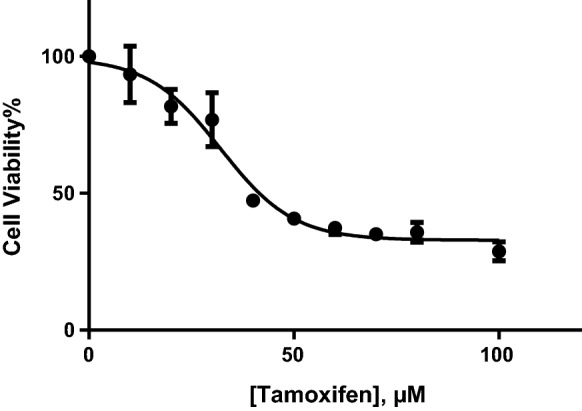
The inhibitory effect of different concentrations of Tamoxifen on the growth of BT474 cells. The results were presented as a percentage relative to the control and graph was plotted using GraphPad Prism 6.01 software

An MTT assay was performed to assess the effectiveness of Tamoxifen from the repurposed drugs in this study on the growth of BT474, the breast cancer stage II, HER2 cell line. Based on the cell survival results, following the treatment with Tamoxifen in different concentrations, the inhibition effect on the cell growth increased with increasing amount of the drug in the culture medium. As it has been shown in Fig. 7, the half maximal inhibitory concentration (IC_50_) of Tamoxifen was 32.13 µM on BT474 cells. It should be noted that the toxic effect of two drugs including, Doxorubicin and Paclitaxel has been proved on MCF-7, SKBR-3 and MCF-7 cell lines, respectively, by other researchers [27–30]. Therefore, we can consider Tamoxifen and other repurposed molecules as effective drugs for breast cancer.

## Conclusion

In this study, a network-based method has been employed for drug repositioning using heterogeneous biological and chemical information. Results show the strength of the proposed method for detecting true drug-disease relationships. RepCOOL suggests four new drugs for breast cancer stage II including Doxorubicin, Paclitaxel, Trastuzumab and Tamoxifen. Structural analysis shows the high structural similarity of these four drugs to the current FDA-approved drugs for breast cancer stage II.

## Data Availability

No applicable.

## Abbreviations

FDA: Food and Drug Administration
DRGN: Drug–gene interaction network
DIGN: Disease-gene interaction network
PPIN: Protein–protein interaction network
GCN: Gene co-expression network
CTD: Comparative Toxic genomics Database
OMIM: Online Mendelian Inheritance in Man
GDAs: Gene–disease associations
NB: Naïve Bayes
RF: Random forest
LR: Logistic regression
DT: Decision tree
SVM: Support vector machine
TP: True positive
TN: True negative
FP: False positive
FN: False negative
ROC: Receiver operator characteristics
AUC: Area under the curve
PCC: Pearson correlation coefficient
MTT: Methyl thiazolyl tetrazolium
FBS: Fetal bovine serum
DMSO: Dimethyl sulfoxide
IC_50_: Half-maximal inhibitory concentration
